# Parents’ depression and anxiety associated with hyperactivity-inattention and emotional symptoms in children during school closure due to COVID-19 in France

**DOI:** 10.1038/s41598-023-31985-y

**Published:** 2023-03-24

**Authors:** Maëva Monnier, Flore Moulin, Marion Bailhache, Xavier Thierry, Stéphanie Vandentorren, Sylvana Côté, Bruno Falissard, Thierry Simeon, Bertrand Geay, Laetitia Marchand-Martin, Marie-Noelle Dufourg, Marie-Aline Charles, Pierre-Yves Ancel, Maria Melchior, Alexandra Rouquette, Cédric Galera, The SAPRIS Study Group, Nathalie Bajos, Fabrice Carrat, Pierre-Yves Ancel, Marie-Aline Charles, Florence Jusot, Claude Martin, Laurence Meyer, Ariane Pailhé, Gianluca Severi, Alexis Spire, Mathilde Touvier, Marie Zins, The ELFE Joint Unit, Xavier Thierry, Thierry Simeon, Bertrand Geay, Laetitia Marie-Noelle Dufourg, Marie-Aline Charles

**Affiliations:** 1 Bordeaux Population Health Research Center, Institut National de la Santé et de la Recherche Médicale (INSERM) U 1219, 146 Rue Léo Saignat, 33077 Bordeaux Cedex, France; 2 Department of Child and Adolescent Psychiatry, Centre Hospitalier Charles Perrens, Bordeaux, France; 3 Pole de Pédiatrie, CHU de Bordeaux, Place Amélie Raba Léon, 33000 Bordeaux, France; 4 Institut National d’Etudes Démographiques (INED), Aubervilliers, France; 5 Inserm, Paris, France; 6 Etablissement Français du Sang (EFS), Paris, France; 7 Santé Publique France, French National Public Health Agency, 94415 Saint-Maurice, France; 8 Research Unit on Children’s Psychosocial Maladjustment, Montreal, QC Canada; 9 Inserm, UVSQ, CESP, Fac. de Médecine – Université Paris-Sud, INSERM 1018, Paris-Saclay University, DevPsy, Villejuif, France; 10 Public Health and Epidemiology Department, AP-HP Paris-Saclay, Bicêtre Hospital, Le Kremlin-Bicêtre, France; 11 Université Paris Cité, Paris, France; 12 Centre for Research in Epidemiology and StatisticS (CRESS), Paris, France; 13 Institut national de recherche pour l’agriculture, l’alimentation et l’environnement (INRAE), Paris, France; 14 Faculté de Médecine St Antoine, Institut Pierre Louis d’Epidémiologie et de Santé Publique (IPLESP), Equipe de Recherche en Épidémiologie Sociale (ERES), Sorbonne Université, Paris, France; 15 IRIS (UMR 8156 CNRS - EHESS - U997 INSERM), Institut de Recherche Interdisciplinaire sur les Enjeux Sociaux - Sciences sociales, politique, santé, Aubervilliers, France; 16 INSERM, Institut Pierre Louis d’Epidémiologie et de Santé Publique (IPLESP), Sorbonne Université, Paris, France; 17 Paris-Dauphine University-PSL, Paris, France; 18 French National Centre for Scientific Research (CNRS), Paris, France; 19 National Institute for Demographic Studies, Paris, France; 20 University of Paris Sud, Paris, France; 21 E4N & EN3-Cohorts, Paris, France; 22 Inra U1125, CNAM, Paris 13 University, Nutritional Epidemiology Research Team (EREN), Bobigny, France; 23 Sorbonne University, Paris, France

**Keywords:** Psychology, Risk factors

## Abstract

Several risk factors of children’s mental health issues have been identified during the pandemic of COronaVIrus Disease first appeared in 2019 (COVID-19). This study aims to fill the knowledge gap regarding the association between parents’ and children’s mental health issues during the COVID-19 school closure in France. We conducted a cross-sectional analysis of data collected in the SAPRIS-ELFE study during the COVID-19 pandemic in France. Using multinomial logistic regressions, we estimated associations between parents’ and children’s mental health issues. Symptoms of anxiety were assessed by the General Anxiety Disorder-7 (GAD-7) and depression by the Patient Health Questionnaire-9 (PHQ-9) for the parents. Hyperactivity/inattention and emotional symptoms in children were assessed by the Strengths and Difficulties Questionnaire (SDQ). The sample included 3496 children aged 8 to 9 years, of whom 50.0% were girls. During the school closure, 7.1% of responding parents had moderate to severe levels of anxiety and 6.7% had moderate to severe levels of depression. A total of 11.8% of the children had an abnormal hyperactivity/inattention score and 6.6% had an abnormal emotional symptoms score. In multivariate regression models, parental moderate to severe level of anxiety and moderate to severe level of depression were associated with abnormal hyperactivity-inattention score (adjusted Odds Ratio (aOR) 3.31; 95% Confidence Interval (CI) 2.33–4.70 and aOR 4.65; 95% CI 3.27–6.59, respectively) and abnormal emotional symptoms score in children (aOR 3.58; 95% CI 2.33–5.49 and aOR 3.78; 95 CI 2.47–5.78 respectively). Children whose parents have symptoms of anxiety and/or depression have an increased likelihood of symptoms of hyperactivity/inattention and emotional symptoms during school closures in France due to COVID-19. Our findings suggest that public health initiatives should target parents and children to limit the impact of such crises on their mental health issues.

## Introduction

During the pandemic of COronaVIrus Disease first appeared in 2019 (COVID-19), family life suddenly changed with the need to adapt to the restrictive collective measures implemented to prevent the spread of the virus^1^. This unexpected situation fraught with incertitude significantly increased the risk of mental health issues both in parents and children^1–9^. Several risk factors for parental mental health issues have been identified, such as having a pre-existing mental health condition, parenting a child with special needs, unemployment, single parenting, gender, and financial difficulties^3,10–12^. COVID-19-related school closures increased the burden on the family, generating a higher risk of parenting-related exhaustion and disrupted parent–child relationships^8,13,14^. In this stressful situation, the mental health issues of parents and children interacted more than ever^4,8^.

Upstream of the COVID-19 context, several studies found an increased risk of psychiatric symptoms in parents of children with psychiatric disorders themselves^15–20^. Some found the same symptoms in parents and children^15,18^. A Dutch study of 1866 children and their parents revealed that parental depressive and Attention Deficit Hyperactivity Disorder (ADHD) symptoms were further predicted by offspring depression and offspring ADHD, respectively^15^. Another study found that parents’ symptoms were not always equivalent to their children’s mental health issues^17^. An Italian case–control study of the parents of 50 children affected by ADHD and of 45 age- and gender-matched healthy children investigated parental psychatric disorders in ADHD children^17^. Compared to parents of children without mental health issues, parents of ADHD children reported higher levels of depression (Odds ratio (OR) 2.36; 95% Confidence Interval (CI) 1.31–4.26) and anxiety disorder (OR 2.01; 95% CI 1.05–3.79)^17^. An American retrospective study of 3- to 7-year-old children with ADHD (n = 98) and non-ADHD children (n = 116) showed a greater prevalence of depression and anxiety in parents of children with ADHD^20^. The same study found that ADHD in the children was associated with higher odds of both maternal and paternal childhood ADHD^20^.

Furthermore, several studies have identified parental mental health issues (e.g. symptoms of anxiety, depression, ADHD) as a predictor of child mental health issues^20–23^. A prospective cohort study identified parental mental health issues as one of the strongest predictors of major depressive disorder in 2519 college students^21^. A recent study of 8002 children aged 9–11 years living in the United States found that after adjustment on age, gender, ethnicity, and environmental factors, greater parent mental health issues (i.e., anxious/depressed, withdrawn, somatic complaints, thought problems, attention problems, aggressive behavior, rule-breaking behavior, and intrusive) was associated with greater child mental health issues (i.e., aggressive behavior, anxiety/depression, attention problems, rule-breaking behavior, somatic complaints, social problems, thought problems, and withdrawn/depressed episodes)^22^.

Although many studies have shown strong associations between parental and child mental health issues, few studies have investigated this relationship during the COVID-19 pandemic, a period of unprecedented impact on the mental health of parents and children^5,6,24–27^. A cross-sectional survey of 1469 parents in the United Arab Emirates found that parents with severe anxiety levels were seven times more likely to report emotional problems in their children^28^. A large-scale cross-sectional population study of 29,202 Hong Kong families with children aged 2–12 years explored child psychosocial wellbeing, parent–child interactions, and parental stress during school closures due to COVID-19^29^. It showed that parents’ mental health issues was positively associated with children’s hyperactivity/inattention and children’s emotional disorder^29^. An Italian survey of 463 parents found that parental stress (assessed with the Parent Stress Scale) was a risk factor of children’s well-being (assessed with the Strengths and Difficulties Questionnaire) during the COVID-19 quarantine^30^. A French cohort study of 432 families found that after adjustment on child’s sex, age, sleeping difficulties, financial difficulties, job situation during lockdown, parents’ symptoms of anxiety-depression were associated with children’s emotional difficulties (OR 5.7; 95% CI 2.4–5.1) and with children’s symptoms of hyperactivity/inattention (OR 2.5; 95% CI 1.4–4.2) during the COVID-19 lockdown^3^.

Our first objective was to estimate the prevalence of anxiety and depressive symptoms in parents as well as hyperactivity/inattention and emotional symptoms in children during the COVID-19 pandemic. Our second objective was to study the association between parents’ mental health issues and children’s during the COVID-19 school closure in France, that might have been highlighted during the pandemic. We hypothesized an increased risk of abnormal hyperactivity/inattention and emotional symptoms in children of parents with a high level of anxiety and/or depression symptoms.

## Methods

**Figure 1:**
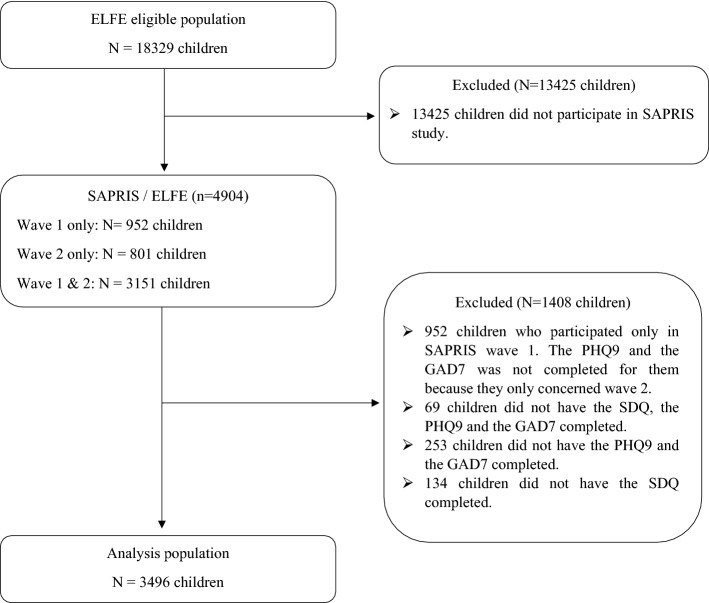
Flowchart of ELFE/SAPRIS cohort during school closure.

This research is based on the SAPRIS (“SAnté, Perception, pratiques, Relations et Inégalités Sociales en population générale pendant la crise COVID-19”) study. SAPRIS was set up to study the main epidemiological, social and behavioral challenges of the COVID-19 pandemic in France in relation to social inequalities in health and healthcare. More details on this project are available elsewhere^31^. SAPRIS is based on a questionnaire sent to participants of five large epidemiological French cohorts. For this research, we used data pertaining to 3496 children aged 8–9 years old in 2020 and participating in ELFE (Etude Longitudinale Française depuis l’Enfance, The French Longitudinal Study since Childhood) population-based birth cohort that focuses on the health of children born in 2011. All parents in the ELFE cohort not lost at follow-up in April 1, 2020, were contacted^31^. The SAPRIS survey does not have any additional inclusion criteria beyond those original to the ELFE cohort (i.e., give birth in one of the 320 French maternal unit, single or twin live births at 33 weeks of gestation, mother 18 years old, no plan to leave metropolitan France within 3 years and informed consent signed)^32^. The entire questionnaire is completed by one of the parents between April 16 and June 21, 2020, during the period of school closure in France due to COVID-19. In this study, 3496 children with complete data on their own and their parents’ mental health were included in this study (see Fig. 1). All methods were carried out in accordance with the relevant guidelines and regulations. Ethical approval and written informed consents were obtained from all subjects and/or their legal guardian. The Inserm ethics evaluation committee (no 2020.04.24 bis_20.04.22.74247, 2020 April 27), and the CNIL (no 920193, 2020 April 30) approved the research.

### Main outcome: children’s mental health issues

Two subscales of the French version of the Strengths and Difficulties Questionnaire (SDQ) were used to assess children’s scores of hyperactivity/inattention and emotional symptoms^33,34^. Five items were used to assess each subscale. Parents were asked to rate each of the items regarding their child for the past 15 days on a three-point Likert scale (0. not true to 2. certainly true). From the five items on each subscale, a global score was calculated from 0 to 10. Regarding hyperactivity/inattention subscale, a score ≤ 5 is considered normal, equal to 6 as borderline, and > 6 as abnormal. For emotional symptoms, a score ≤ 3 is considered normal, equal to 4 as borderline, and > 4 as abnormal.

### Main exposure: parents’ mental health issues

Two measures of parental mental health issues were collected. The first assessed symptoms of anxiety through the Generalized Anxiety Disorder-7 (GAD-7)^35,36^. This is a 7-item scale scored 0 to 3 (0. never, 1. several days, 2. more than half the time or 3. almost every day), providing a 0 to 21 severity score. Responses were classified as follows: absence of anxiety (< 4), mild anxiety (≤ 9), moderate to severe anxiety (> 9).

A second measure assessed depressive symptom severity with the Patient Health Questionnaire-9 (PHQ-9)^37,38^. This module includes 9 items on which the subject estimates the frequency of depressive symptomatology (0. never, 1. several days, 2. more than half the time or 3. almost every day). The total PHQ-9 score varies between 0 and 27. Levels of depression are categorized as follows: minimal (score < 5), mild (< 10), moderate to severe (≥ 10)^39^.

### Covariates

Several covariates based on the literature review were taken into account in the statistical analyses, including the child’s sleeping difficulties (yes *vs* no), child’s sex (male *vs* female), sex of the responding parent (male *vs* female), if the child lived with both parents (yes *vs* no), parents’ occupational grade (executive, intermediate and executive, intermediate and employee, independent, laborer, both inactive or only one employee/laborer), household income per month before lockdown in euros (< 2000, [2000; 4000[≥ 4000), and perceived financial situation since the lockdown (stable *vs* declining).

### Statistical analysis

Chi-square tests were used to compare the characteristics of the ELFE/SAPRIS cohort children included vs not included in this study. Descriptive statistics (percentages) were used to describe the sample demographic characteristics. Classification and Regression Tree methods (CART) were used to perform multiple imputation to handle missing data^40^. CART methods are useful for imputation because they are robust against outliers, can be applied to mixed data (both continuous and categorical), and can deal with multicollinearity^40,41^. To test whether parents’ mental health influenced their children’s mental health, we conducted unadjusted and adjusted multinomial logistic regressions, with hyperactivity/inattention symptoms or emotional symptoms as the dependent variable and depressed or anxious status of parents as predictors. Multinomial logistic regression models were adjusted on the child’s emotional symptoms or hyperactivity/inattention symptoms, sleeping difficulties sex, on the respondent’s sex, if the child lived with both parents, the parents’ occupational category, household income per month before lockdown and perceived financial situation since the lockdown.

Data were analyzed using the *nnet* package in* R* (*version 3.6.1*) with multinomial logistic regressions specified using the *multinom* function. The *mice* package and its *cart* method were used to perform multiple imputation. The following variables were imputed: child’s sex (0.77% of NA), household income per month before lockdown (24.63% of NA), perceived financial situation since the lockdown (4.52% of NA), child’s sleeping difficulties (0.11% of NA), and parents’ occupational category (0.83% of NA).

## Results

Table 1 shows the characteristics of the ELFE/SAPRIS cohort children who were included or not in this study. The two populations did not differ significantly on most variables. They differed only in the number of children living with both parents and in the parents’ occupational category. There was a lower proportion of children living with both parents as well as children of parents of high occupational grade. The association between the mental health issues of parents and children might even more pronounced in the population of non-included children.

**Table 1:** Comparison of characteristics of children in cohort SAPRIS/ELFE included vs. excluded in this study (n = 4904).

	Children included (n = 3496)	Children excluded (n = 1408)	p-value
	n (%)	n (%)	
Child’s sex	3469 (99.2)	1395 (99.1)	0.93
Female	1736 (50.0)	700 (50.2)	
Male	1733 (50.0)	695 (49.8)	
Sex of responding parent	3496 (100.0)	1376 (97.7)	0.26
Female	3211 (91.8)	1277 (92.8%)	
Male	285 (8.2)	99 (7.2%)	
Child’s sleeping difficulties	3492 (99.9)	1239 (88.0)	0.39
Yes	1349 (38.6)	496 (40.0)	
No	2143 (61.4)	743 (60.0)	
Child lives with both parents	3496 (100.0)	1387 (98.5%)	< 0.05*
Yes	3113 (89.0)	1122 (80.9)	
No	383 (11.0)	265 (19.1)	
Parents’ occupational category	3467 (99.2)	1355 (96.2)	< 0.05*
Executive	1145 (33.0)	388 (28.6)	
Intermediate and executive	953 (27.5)	372 (27.5)	
Intermediate and employee	818 (23.6)	334 (24.6)	
Independent	297 (8.6)	121 (8.9)	
Laborer	183 (5.3)	90 (6.6)	
Both inactive or only one employee/laborer	71 (2.0)	50 (3.7)	
Household income per month before lockdown (in euros)	2635 (75.4)	217 (15.4)	0.66
< 2000	183 (6.9)	15 (6.9)	
[2000; 4000[	1150 (43.6)	88 (40.6)	
≥ 4000	1302 (49.4)	114 (52.5)	
Perceived financial situation since lockdown	3338 (95.5)	1217 (86.4)	0.25
Declining	910 (27.3)	353 (29.0)	
Stable or increasing	2428 (72.7)	864 (71.0)	
Child’s mental health issues			
Hyperactivity/inattention	3496 (100.0)	1153 (81.9)	0.30
Abnormal	411 (11.8)	150 (13.0)	
Borderline	306 (8.8)	88 (7.6)	
Normal	2779 (79.5)	915 (79.4)	
Emotional symptoms	3496 (100.0)	1171 (83.2)	0.22
Abnormal	231 (6.6)	94 (8.0)	
Borderline	196 (5.6)	60 (5.1)	
Normal	3069 (87.8)	1017 (86.8)	
Mental health issues of responding parent			
Anxiety	3496 (100.0)	214 (15.2)	0.55
Moderate to severe	247 (7.1)	18 (8.4)	
Mild	831 (23.8)	45 (21.0)	
Minimal	2418 (69.2)	151 (70.6)	
Depression	3496 (100.0)	188 (13.4)	0.46
Moderate to severe	234 (6.7)	12 (6.4)	
Mild	919 (26.3)	42 (22.3)	
Minimal	2343 (67.0)	134 (71.3)	

Table 2 shows the characteristics of the 3496 children included in the study. Of these children, 50% are girls (1736) and 89% (3113) lived with both parents during lockdown. 11.8% (411) of the children had an abnormal hyperactivity/inattention score and 6.6% (231) had an abnormal emotional symptoms score during the school closures.

**Table 2:** Characteristics of children and their families in the ELFE cohort of the SAPRIS study during school closure in France (n = 3496).

	n (%)
Child’s sex	3469 (99.2)
Female	1736 (50.0)
Male	1733 (50.0)
Sex of responding parent	3496 (100.0)
Female	3211 (91.8)
Male	285 (8.2)
Child’s sleeping difficulties	3492 (99.9)
Yes	1349 (38.6)
No	2143 (61.4)
Child lives with both parents	3496 (100.0)
Yes	3113 (89.0)
No	383 (11.0)
Parents’ occupational category	3467 (99.2)
Executive	1145 (33.0)
Intermediate and executive	953 (27.5)
Intermediate and employee	818 (23.6)
Independent	297 (8.6)
Laborer	183 (5.3)
Both inactive or only one employee/laborer	71 (2.0)
Household income per month before lockdown (in euros)	2635 (75.4)
< 2000	183 (6.9)
[2000; 4000[	1150 (43.6)
≥ 4000	1302 (49.4)
Perceived financial situation since lockdown	3338 (95.5)
Declining	910 (27.3)
Stable or increasing	2428 (72.7)
Child’s mental health issues	
Hyperactivity/inattention	3496 (100.0)
Abnormal	411 (11.8)
Borderline	306 (8.8)
Normal	2779 (79.5)
Emotional symptoms	3496 (100.0)
Abnormal	231 (6.6)
Borderline	196 (5.6)
Normal	3069 (87.8)
Mental health issues of responding parent	
Anxiety	3496 (100.0)
Moderate to severe	247 (7.1)
Mild	831 (23.8)
Minimal	2418 (69.2)
Depression	3496 (100.0)
Moderate to severe	234 (6.7)
Mild	919 (26.3)
Minimal	2343 (67.0)

Most parents (91.9%) who participated were mothers (n = 3211). Among responding parents, 7.1% (247) had moderate to severe anxiety, and 6.7% (234) had moderate to severe depression (Table 2).

### Parent’s mental health issues and children’s hyperactivity/inattention

Table 3 shows the adjusted and unadjusted associations between parents’ mental health issues and children’s symptoms of hyperactivity/inattention. Having parents with moderate to severe anxiety or mild anxiety during school closures was associated with an increased risk of an abnormal hyperactivity/inattention score in children (OR 4.89; 95% CI 3.53–6.76 and OR 2.87; 95% CI 2.27–3.61, respectively) as well as a borderline score (OR 2.51; 95% CI 1.66–3.78 and OR 1.94; 95% CI 1.49–2.52, respectively). These associations remained statistically significant in the multivariate regression model. Adjusted on covariates, moderate to severe anxiety and mild anxiety in the responding parent during school closures were associated with an abnormal hyperactivity-inattention score (aOR 3.31; 95% CI 2.33–4.70 and aOR 2.25; 95% CI 1.76–2.88, respectively) and its borderline level (aOR 2.18; 95% CI 1.42–3.34 and aOR 1.81; 95% CI 1.38–2.37 respectively).

**Table 3:** Association between parents’ mental health issues and children’s hyperactivity/inattention score: multinomial logistic regressions (n = 3496).

	OR		aOR ^2^	
	Abnormal^1^	Borderline^1^	Abnormal^1^	Borderline^1^
Anxiety of responding parent				
Moderate to severe	4.89 [3.53; 6.76]	2.51 [1.66; 3.78]	3.31 [2.33; 4.70]	2.18 [1.42; 3.34]
Mild	2.87 [2.27; 3.61]	1.94 [1.49; 2.52]	2.25 [1.76; 2.88]	1.81 [1.38; 2.37]
Minimal	–	–	–	–
Depression of responding parent				
Moderate to severe	6.99 [5.07; 9.65]	2.29 [1.44; 3.64]	4.65 [3.27; 6.59]	2.00 [1.24 ; 3.23]
Mild	3.21 [2.54; 4.05]	2.48 [1.93; 3.19]	2.54 [1.99; 3.25]	2.26 [1.74 ; 2.93]
Minimal	–	–	–	–

^1^Normal is reference group.

^2^Results adjusted on child’s emotional symptoms score, child’s sleeping difficulties, child’s sex, sex of responding parent, if the child lived with both parents, parents’ occupational category, household income per month before lockdown and perceived financial situation since lockdown.

Furthermore, having parents with moderate to severe depression or mild depression during school closure was associated with an increased likelihood of abnormal hyperactivity/inattention scores (OR 6.99; 95% CI 5.07–9.65 & OR 3.21; 95% CI 2.54–4.05, respectively) in children; the same was observed for borderline scores (OR 2.29; 95% CI 1.44–3.64 & OR 2.48; 95% CI 1.93–3.19, respectively) (Table 3). These results remained significant in the multivariate analysis. Adjusting on covariates, moderate to severe parental depression and mild depression during school closures were associated with abnormal hyperactivity-inattention scores (aOR 4.65; 95% CI 3.27–6.59 & aOR 2.54; 95% CI 1.99–3.25, respectively) and borderline scores in children (aOR 2.00; 95% CI 1.24–3.23 & aOR 2.26; 95% CI 1.74–2.93, respectively).

### Parents’ mental health issues and children’s emotional symptoms

Table 4 shows the adjusted and unadjusted associations between parents’ mental health issues and children’s emotional symptoms scores. Having a parent with moderate to severe anxiety or mild anxiety during school closure was associated with an increased risk of an abnormal emotional symptoms score in children (OR 5.81; 95% CI 3.98–8.61 and OR 3.50; 95% CI 2.59–4.72, respectively) as well as its borderline score (OR 3.11; 95% CI 1.95–4.95 and OR 2.38; 95% CI 1.73–3.27, respectively). Adjusting on covariates, moderate to severe anxiety and mild anxiety in the responding parent during school closures remained associated with an abnormal emotional symptoms score (aOR 3.58; 95% CI 2.33–5.49 and aOR 2.52; 95% CI 1.84–3.46, respectively) and its borderline score (aOR 2.12; 95% CI 1.30–3.46 and aOR 1.98; 95% CI 1.43–2.75, respectively).

**Table 4:** Association between parents’ mental health issues and children’s emotional symptoms score: multinomial logistic regressions (n = 3496).

	OR		aOR^2^	
	Abnormal^1^	Borderline^1^	Abnormal^1^	Borderline^1^
Anxiety of responding parent				
Moderate to severe	5.81 [3.98; 8.61]	3.11 [1.95; 4.95]	3.58 [2.33; 5.49]	2.12 [1.30; 3.46]
Mild	3.50 [2.59; 4.72]	2.38 [1.73; 3.27]	2.52 [1.84; 3.46]	1.98 [1.43; 2.75]
Minimal	–	–	–	–
Depression of responding parent				
Moderate to severe	6.69 [4.56; 9.80]	2.62 [1.58; 4.34]	3.78 [2.47; 5.78]	1.79 [1.05; 3.05]
Mild	2.77 [2.05; 3.74]	2.22 [1.63; 3.03]	1.98 [1.44; 2.73]	1.78 [1.29; 2.46]
Minimal	–	–	–	–

^1^Normal score is reference group.

^2^Results adjusted on child’s hyperactivity/inattention score, child’s sleeping difficulties, child’s sex, sex of responding parent, if the child lived with both parents, parents’ occupational category, household income per month before lockdown and perceived financial situation since lockdown.

Having parents with mild to severe depression during school closures was associated with an increased risk of an abnormal emotional symptoms score in children (OR 6.69; 95% CI 4.56–9.80 and OR 2.77; 95% CI 2.05–3.74, respectively) as well as its borderline score (OR 2.62; 95% CI 1.58–4.34 and OR 2.22; 95% CI 1.63–3.03, respectively) (Table 4). Adjusting on covariates, moderate to severe depression and mild depression in the responding parent during school closures remained associated with an abnormal emotional symptoms score (aOR 5.84; 95% CI 3.91–8.72 and aOR 2.70; 95% CI 1.98–3.68, respectively) and its borderline score (aOR 2.47; 95% CI 1.47–4.15 and aOR 2.28; 95% CI 1.66–3.13, respectively).

## Discussion

This study shows that moderate to severe anxiety and moderate to severe depression in parents during school closures were associated with both abnormal hyperactivity-inattention and emotional symptoms scores in children. Furthermore, mild anxiety and mild depression in the responding parent during school closures were associated with abnormal scores of both hyperactivity-inattention and emotional symptoms in children.

Although the impact of COVID-19 on the decline of mental health remains debated up to date^42–44^, our results are consistent with previous research showing that parental mental health issues during the pandemic were associated with children’s mental health issues^3,8,45^. A longitudinal Canadian study of 68 families of children aged 7 to 9 years showed that higher levels of maternal anxiety during the pandemic were associated with a greater increase in internalizing problems in children^8^. A cross-sectional Italian study of 721 parents found that parents’ behaviors during lockdown (i.e., employment status) were significantly related to their own internalizing symptoms. These symptoms, in turn, had a strong and positive predictive effect on children’s internalizing problems^45^.

The cross-sectional associations can be explained by the reciprocal influences of parental and child mental health issues. This study shows that the associations between parents’ and children’s mental health issues which haves already been highlighted outside the COVID context^15–20^ is found to be also present during the COVID-19 pandemic. Based on the results of this study, we are not able to know whether this association is stronger during the COVID-19 period due to the lack of data prior to the pandemic. However, the lockdown and school closures have impacted families’ daily life and have challenged their parenting skills. Dealing with school closure and the lockdown is a particularly stressful situation for parents who have to balance personal life, work, and ensure the continuity of children's education and learning^1^. This situation puts parents at a higher risk of experiencing anxiety, stress, depression, potentially impairing their ability to be supportive caregivers^1^. We speculate that more anxiety and depression in parents prevents them from understanding their child’s needs and responding appropriately. Moreover, the COVID-19 pandemic was marked by an increase in irritability, shouting at children, verbal abuse, and parents punishing children^46–48^. Such behaviors are associated with an increased risk of disorders in children^49–51^. On the other hand, managing children with hyperactivity/inattention and/or emotional symptoms can be a source of stress and mental distress for parents^14,52^. An Italian study of 1226 parents showed that having a child with cognitive or physical disabilities was a risk factor of parenting-related exhaustion during COVID-19 lockdown^14^. Furthermore, a recent cohort study of 917 parents showed that more child emotional and hyperactivity-inattention symptoms were significant predictors of parental distress during the lockdown^52^. Despite these environmental factors that may explain the parents’ and children’s mental health issues mutually influencing each other, the hypothesis of a genetic vulnerability cannot be ruled out. Indeed, several studies produced evidence for genetic overlap between ADHD and bipolar disorder^53,54^, and ADHD and major depressive disorder^53–55^. Studies taking into account both environmental risk factors and genes are needed to better understand the relationships between various mental health issues in parents and offspring.

Our research has several strengths. First, the sample was large with sufficient power, thus increasing the likelihood of obtaining more conclusive results with adjustments. In addition, the measures of parental and children’s mental health were based on validated scales with good psychometric qualities. Moreover, we took several confounding factors into account such as the sex of the child and the responding parent, the occupational category of the household and the perceived financial situation.

However, several limitations must be taken into consideration. First, we were not able to assess a potential effect of school closures due to the absence of repeated measures of children’s and parents’ mental health issues before the lockdown. Due to the lack of measurement of parents’ and children’s mental health issues just prior to the pandemic, we are unable to identify an effect of the pandemic on the parents’ and children’s mental health issues association. On the other hand, this study focuses on children aged 8–9 years; other studies are needed on other age groups to be able to generalize the results to other age ranges. In addition, we were unable to infer any causal relationship between parents’ and children’s mental health issues. Also, the sample was composed mostly of mothers. However, we adjusted the analyses for the gender of the responding parent to make the results generalizable. Furthermore, we didn’t have a measure of parental stress, which is an essential variable when studying parents’ mental health issues, especially during the period of COVID-19^30^. At last, these are only self-reported measures and thus are subject to bias in the assessment. To avoid the later, children’s mental health issues should be measured by a health care professional. Parents who are anxious or depressed may be more likely to indicate a mental disorder in their child than healthy parents.

For future research, we recommend to have repeated measures and study a wider range of mental health issues in parents (e.g., mood disorders, substance use disorders, and psychotic disorders) and children (e.g., learning disabilities, autism spectrum disorders). It could also be interesting to study how the association between parents’ and children’s mental health issues evolves once the school takes over.

The present findings have significant implications. Parenting while having a mental health issues during school closure may represent a threat to children’s mental health issues and have an impact on the way children and parents interact. Governments and healthcare authorities should be alerted to the effects that lockdown and school closure may worsen symptoms in children and their parents. Whenever mental health issues are suspected in the child or their parents, it is of utmost importance that clinicians take into consideration the mental health of the other family members close to the patient. In the same line, prevention strategies promoting parental mental health issues could have the potential for positive long-term impacts on child mental health issues.

Longitudinal studies are now needed to assess changes in the mental health of children and parents during and after the pandemic. These results underline the importance of focusing on mental health issues in children in the presence of parental mental health issues and vice-versa. This seems to be even truer in this period of collective stress and promiscuity characteristic of school closures and the lockdown in France due to the COVID-19 pandemic. In addition, continuing to deliver care during the crisis has posed problems. One of our previous studies showed that 87% of children receiving psychological care prior to the pandemic were unable to continue to receive it^56^. More thorough follow-up of children and parents at risk is required to ensure continuity of care during such periods of risk. Finally, the creation of a national cohort would allow retrospective and prospective data to be gathered to study the evolution of mental health in France over time and during crises such as the COVID-19 pandemic.

## Conclusion

Children of parents with anxiety and/or depression were at increased risk of hyperactivity/inattention and emotional symptoms during school closures in France due to COVID-19. The COVID-19 pandemic and associated school closures have caused disruption to family life and affected the wellbeing of children and their parents. Findings suggest that public health initiatives should target parents and children to limit the impact of such crises on their mental health issues.

## Data Availability

The study data are protected by the health data privacy regulations set out by the French National Commission on Informatics and Liberty (Commission Nationale de l’Informatique et des Libertés, CNIL). The data are available upon reasonable request after consultation with the Sapris steering committee. While French law forbids free access to the Sapris data, it may be granted by the steering committee after legal verification of how the data will be used.The datasets generated during and/or analysed during the current study are available from Dr Cédric Galéra on reasonable request.
